# The volumetric relationship of mastoid cavity and paranasal sinuses in unilateral chronic otitis media

**DOI:** 10.1007/s11845-026-04283-5

**Published:** 2026-02-28

**Authors:** Elif Kaya Çelik, Kemal Keseroğlu, Mert Nahir, Hilal Irmak Sapmaz, Zehra Gök, Tugce Pütürgeli Özer, Güleser Saylam

**Affiliations:** 1 Faculty of Medicine. Department of Otolaryngology Head and Neck Surgery, Tokat Gaziosmanpaşa University, Tokat, Turkey; 2 Private Clinic, Ankara, Turkey; 3 Faculty of Medicine. Department of Anatomy, Tokat Gaziosmanpaşa University, Tokat, Turkey; 4 Faculty of Medicine. Department of Otolaryngology Head and Neck Surgery, İstinye University, Istanbul, Turkey; 5 Faculty of Medicine. Department of Otolaryngology Head and Neck Surgery, Lokman Hekim University, Ankara, Turkey

**Keywords:** 3D imaging, Chronic otitis media, Mastoid pneumatization, Paranasal sinuses

## Abstract

**Aims:**

A number of studies link ear and paranasal sinus disorders even though correlation between the development of the paranasal sinuses and the mastoid has not been demonstrated clearly. In this study, we examined temporal bone computed tomography (CT) scans to calculate mastoid and paranasal sinus volumes using three-dimensional (3D) reconstruction in patients with unilateral nonsuppurative chronic otitis media (COM) and investigated whether there was a correlation between mastoid and paranasal sinus volumes.

**Methods:**

Of 85 patients with unilateral nonsuppurative COM, 51 patients who underwent unilateral type 1 tympanoplasty and has temporal bone CTs were included in the study. Open-access Horos DICOM Viewer software (Horos Project, Geneva, Switzerland) was used to measure the volumes of sphenoid, maxillary and frontal sinuses and mastoid air cells. Maxillary, frontal, sphenoid sinus and mastoid air cell volumes were calculated and analyzed.

**Results:**

Unilateral nonsuppurative chronic otitis media caused a statistically significant decrease in ipsilateral mastoid pneumatization, causing an asymmetry between two mastoid air cell systems. Ipsilateral frontal and maxillary sinus volumes were decreased and ipsilateral sphenoid sinus volume increased, however these findings were not statistically significant.

**Conclusion:**

Middle ear pathologies cause mastoid cell hypopneumatization. Care should be taken as these changes may also have an effect on the paranasal sinuses. Existing pathologies should be taken into consideration before paranasal or mastoid surgery to avoid possible complications.

## Introduction

All mammals have ventilation systems in their skulls with functions of warming and humidifying inhaled air, reducing the weight of the skull and controlling the amount of inhaled air, regulating resonance during phonation and in humans [1]. The paranasal sinuses (PNS) and the mastoid air cell system are the best-defined ventilation structures in humans and they become pneumatized gradually [2]. The mastoid antrum reaches its adult size by the 34th week of gestation. Temporal bone pneumatization is completed around puberty with the development of the last air cells in the petrous apex [3].

The volume of mastoid air cells and paranasal sinuses have been investigated in a number of studies [2, 4]. Mastoid air cells (MAC) and paranasal sinuses are in contact with each other and go through similar pneumatization stages during their development, however the interaction between them has not yet been clarified. Many factors, including environmental factors, genetic disorders and past infections may affect the pneumatization process, which is their main distinguishing characteristic.

Some hypotheses claim that the degree of mastoid pneumatization depends on genetics or the degree of pathological inheritance in childhood [5]. Heredity is considered as a common factor in the pneumatization process in both hypotheses, therefore, the second hypothesis may better explain the differences among the individuals. Pneumatization of mastoid air cells may be promoted by applying positive pressure to the nasopharynx via the Eustachian tube, and this also applies to the pneumatization of the PNS [6].

To date, various methods have been used to evaluate the volume of air spaces in human body. Volumetric analyzes have been employed using various CT reconstruction techniques in a number of studies. 3D CT reconstructions provide excellent evaluation of the nasal and paranasal sinus cavities, and precise measurement techniques provide the opportunity to assess the factors affecting them. The effect of paranasal sinus pathologies on mastoid pneumatization and their association with middle ear disorders have been the subject of many studies [7]. We could not find any studies in the literature that investigated the effect of chronic otitis media (COM) on the volume of the paranasal sinuses. In our study, we aimed to measure mastoid pneumatization in patients diagnosed with unilateral COM and investigate its relationship with the volume of the paranasal sinuses.

## Methods

For this retrospective and descriptive study, 85 patients who underwent unilateral type 1 tympanoplasty with the diagnosis of unilateral COM in the Otorhinolaryngology Clinic of a tertiary Training and Research Hospital between May 2017 and July 2021 were selected as the candidates. Nonsuppurative otitis media was defined as cases confirmed by otoscopic findings and audiological evaluation, with no otorrhea for at least 3 months.The local ethics committee approved the study protocol (19.04.2021–109/16). The patients whose paranasal sinuses were not fully included in the preoperative temporal bone CT were excluded, and 51 patients were included in the study. The side with COM was accepted as the study side, and the side of the healthy ear was considered as control. Mastoid, frontal, maxillary and sphenoid sinus volumes were measured on the study side. Contralateral mastoids and contralateral paranasal sinus volumes of the same patients were included in the study as the control group.

The exclusion criteria were being under 18 years of age and having a history of middle ear disease/mastoid surgery in the opposite ear. The patients with chronic rhinosinusitis and/or nasal polyps, a history of previous middle ear or paranasal sinus surgery, and CT findings consistent with bilateral COM and/or mastoiditis were also excluded from the study.

Frontal sinus volume was defined as the sinus volume above the level of the agger nasi cell. Mastoid volume was defined as the sum of the volumes of air cells behind the aditus ad antrum.

### Image acquisition and volume analysis

**Fig. 1:**
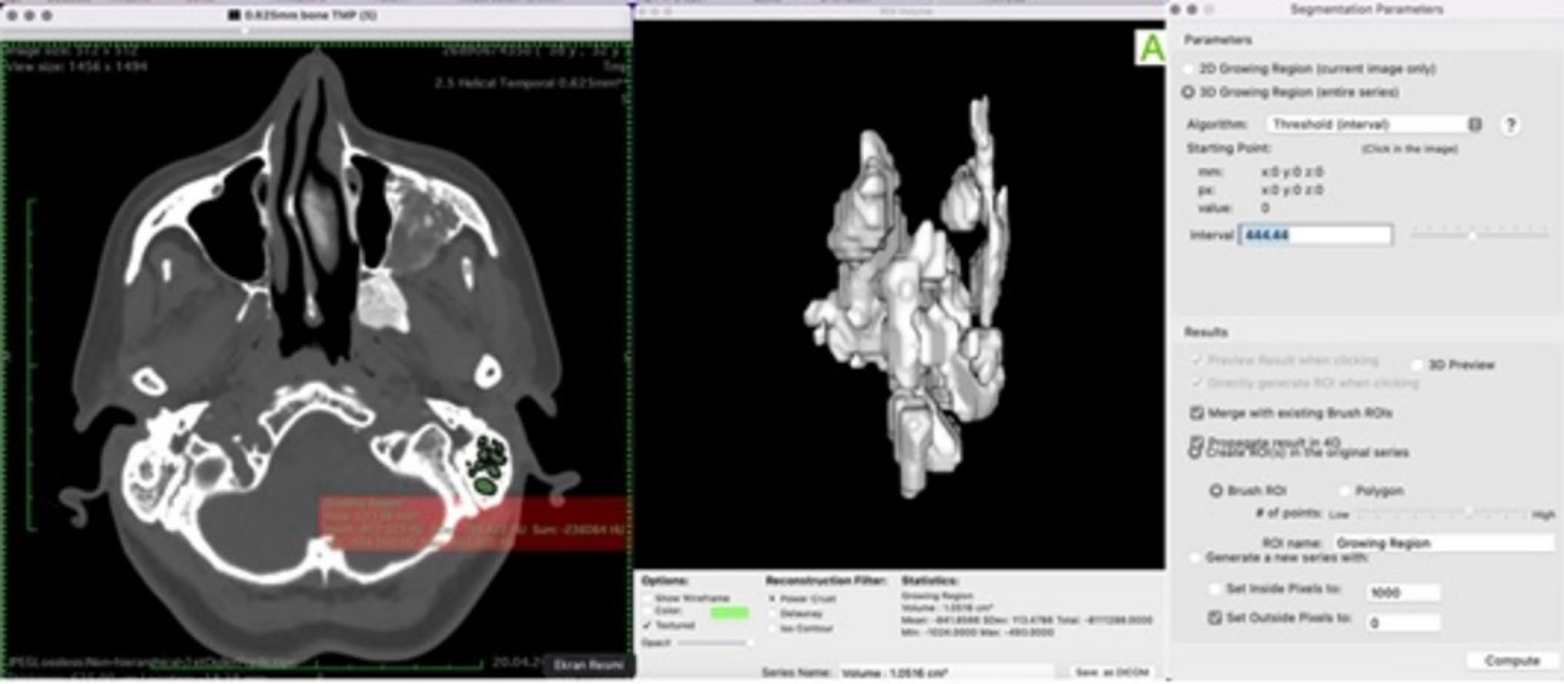
Procedure steps of measurement of mastoid pneumatization. Axial slice of the computed tomography scan of mastoid cells

**Fig. 2:**
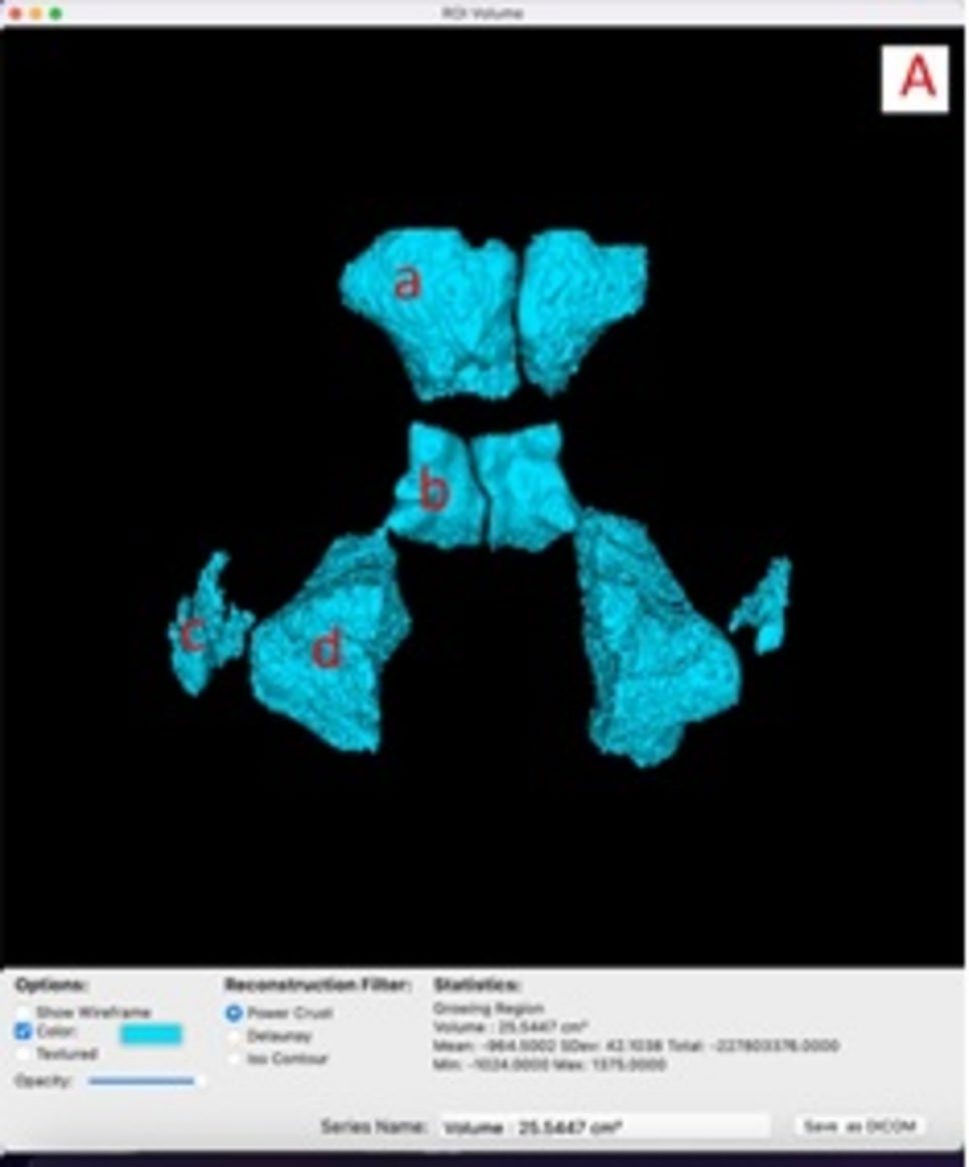
Three-dimensional reconstruction by surface rendering. Pneumatization of the paranasal sinuses and mastoid cells. Front view a) Frontal sinus, b) sphenoid sinus, c) maxillary sinus, d) mastoid cells

**Fig. 3:**
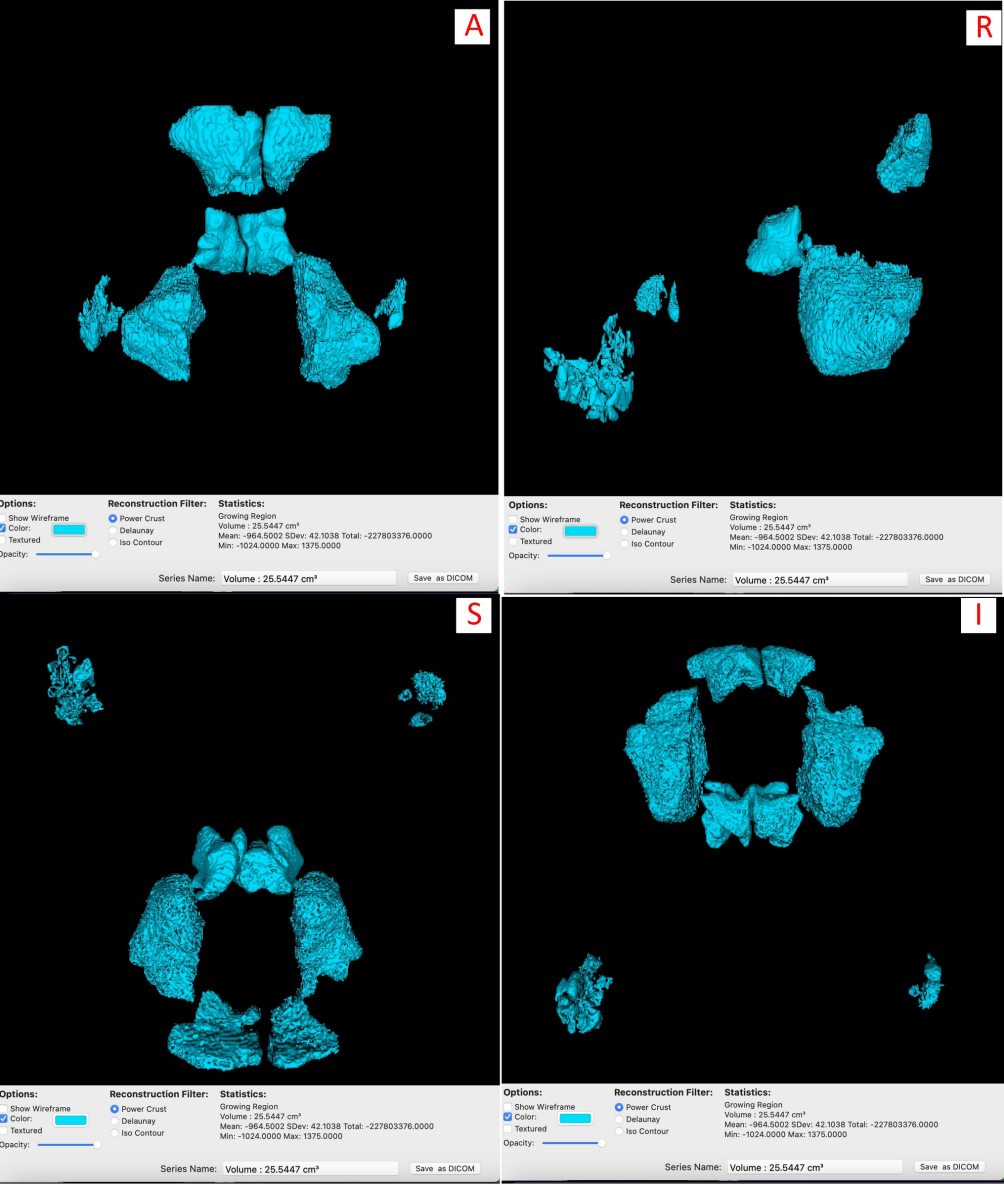
Multi-angle views of the three-dimensional surface rendering reconstruction. A-. Front view, R-Lateral view, S-Superior view, I-Inferior view

Open-access Horos DICOM Viewer software (Horos Project, Geneva, Switzerland) was utilized for measuring the volumes of sphenoid, maxillary and frontal sinuses and mastoid air cells. Anonymized CT image series with a slice thickness of 0.625 mm were uploaded to the software. The contrast and brightness of the images were adjusted to “bone” setting. Mastoid cells were identified by the researcher (Fig. 1). The identified voids were marked using the “Grow region” tool located in the ROI segment. All volumetric measurements were performed in duplicate by the same observer with a one-week interval, showing excellent intraobserver reliability (ICC = 0.92). The accuracy of the markings was verified by the researcher at this stage, and any deficiencies were rectified. Subsequently, the “Compute Volume” option in the “ROI Volume” segment was employed to automatically identify cells present in each slice and to obtain volume data. The procedure steps were repeated for maxillary, sphenoid and frontal sinuses (Figures 2 and 3).

### Statistical analysis

In study, data for continuous variables were presented as mean ± standard deviation or median and first and third quartiles. Shapiro–Wilk’s test was used to assess the normal distribution of data. Independent samples t test was used to compare the normally distributed independent variables between two groups, and Mann–Whitney U test was used to compare the non-normally distributed independent variables between two groups. A post-hoc power analysis was conducted using G*Power software. The analysis showed a power of 0.82 for detecting significant differences in mastoid volumes between groups (α = 0.05, effect size d = 0.65). The statistical significance level was set at p < 0.05. Statistical analyses were performed using a commercial software (IBM SPSS Statistics for Windows, Version 27.0. Armonk, NY: IBM Corp).

## Results

The mean age of 51 patients included in the study was 36.53 ± 11.08 years. Among all, 64.7% of the patients were male and 35.3% were female. The mean age of men was 36.06 ± 10.3 years, and the mean age of women was 36.79 ± 11.62 years (p = 0.824).

The volumes of a total of 408 cavities made calculated including mastoid air cells and frontal, sphenoid and maxillary sinuses. The mean sphenoid sinus volume was 9.59 ± 5.7 cm^3^ in men and 5.97 ± 3.5 cm^3^ in women (p = 0.024) (Table 1).

**Table 1:** Distribution of quantitative variables by gender

Variables	Total	Gender		p
		Male *(n* = *18)*	Female *(n* = *33)*	
Age(year)	36,53 ± 11,08	36,06 ± 10,3	36,79 ± 11,62	0,824
Maxillar Sinus(cm^3^)	19,15 ± 9.0	21,2 ± 10,85	18,04 ± 7,78	0,233
Mastoidea(cm^3^)	1,92 [0,93–2,8]	2,04 [1,19–3,66]	1,92 [0,83–2,58]	0,110*
Frontal sinus(cm^3^)	3,93 [2,1–5,76]	5,45 [3,06–6,41]	3,48 [2,1–4,67]	0,129*
Sphenoid sinus(cm^3^)	6,23 [4,51–8,46]	8,26 [5,51–13,65]	5,93 [3,87–6,97]	0,024*

*Data are shown as mean* ± standard deviation or median [Q1-Q3]*: Mann Whitney U test was used. Independent Samples t test was used for the others

The mastoid volume was 0.9 cm^3^ [0.55–1.77] on the side with COM, and 1.92 cm^3^ [0.93–2.8] on the control side, and the difference between the two sides was statistically significant (p = 0.001) (Table 2). The mean maxillary sinus volume was 18.52 ± 8.72 cm^3^ on the COM side and 19.15 ± 9.0 cm^3^ on the control side (p = 0.718).

**Table 2:** Distribution of quantitative variables by group

Variables(cm^3^)	Total *(n* = *102)*	Group		p
		Control *(n* = *51)*	Patient *(n* = *51)*	
Maxillar Sinus	18,84 ± 8,82	19,15 ± 90	18,52 ± 8,72	0,718
Mastoidea	1,24 [0,73–2,28]	1,92 [0,93–2,8]	0,9 [0,55–1,77]	< 0,001*
Frontal sinus	3,91 [2,1–6,41]	3,93 [2,1–5,76]	3,89 [1,99–7,3]	0,883*
Sphenoid sinus	6,4 [3,46–9,03]	6,23 [4,51–8,46]	6,62 [3,12–9,95]	0,698*

*Data are shown as mean* ± standard deviation or median [Q1-Q3]*: Mann Whitney U test was used. Independent Samples t test was used for the others

The mean frontal sinus volume was 3.89 cm^3^ [1.99–7.3] on the COM side and 3.93 cm^3^ [2.1–5.76] on the control side (p = 0.883) (Table 2).

The mean sphenoid sinus volume was 6.62 cm^3^ [3.12–9.95] on the COM side and 6.23 cm^3^ [4.51–8.46] on the control side (p = 0.698).

## Discussion

The development paranasal sinuses begins in 3-4th months of gestation and they reach their adult size at different ages [8]. There are two active pneumatization phases during paranasal sinus development, the first between birth and 4 years of age and the second between 8 and 12 years of age [9]. Paranasal sinus anatomy is quite complex and varies among individuals. The development of various CT-based volumetric measurements has replaced traditional methods, and the volumes and anatomical relationships of the paranasal sinuses may now be calculated with a number of methods [4]. With the increasing interest in 3D CT models, studies have focused on defining the “normal” paranasal sinus volumes of the general population. These studies also explored the physiological and pathological factors that influence paranasal sinus development. In paranasal sinus surgery, anatomical knowledge and variations are essential to avoid complications of surgery. Care should be taken in terms of developmental or secondary changes in both temporal bone and paranasal sinus surgery. For this purpose, 2D/3D paranasal sinus volume investigations were performed in many studies, and the factors influencing pneumatization were investigated [10, 11].

Mastoid pneumatization is of critical importance for surgery, and the factors leading to hypopneumatization in the mastoid bone remain a subject that requires further in-depth investigation. The studies that evaluated mastoid pneumatization in normal mastoids reported similar pneumatization on the right and left sides^1^ (1). However, hypopneumatization is obvious in diseased ears [12]. Whether hypopneumatization causes chronic otitis or infection causes hypopneumatization is not yet clear. The airspace systems, comprising both the PNS and the MAC, are formed through the resorption of the epithelium in the mastoid bone and the paranasal sinuses. Considering the similar developmental processes of both structures, it is thought that, in addition to genetic factors, the airflow in the nasal airway may also influence pneumatization. The evaluation of paranasal sinus volumes may provide further insight into this relationship.

In this study, we focused on mastoid pneumatization, its volumetric relationship with the paranasal sinuses, and the asymmetry in the right and left mastoid cells using 3D CT reconstruction. When we compared the side with COM with the opposite side, mastoid pneumatization was significantly less on the side with COM. Detection of this condition is important before surgery, and the effect of mastoid hypopneumatization on anatomical and functional results is also an important area of research. Baklacı et al. reported that mastoid and middle ear volumes affected surgical success in pediatric population [13]. In our study, hypopneumatization was not significant in ipsilateral paranasal sinuses. If we had detected hypopneumatization in the ipsilateral PNS, we could have concluded that the mastoid and paranasal sinus developments might be correlated, and based on this, we could even comment that hypopneumatization may cause COM.

Thomas and colleagues were the first to analyze air-containing structures, showing that there was no correlation between PNS and mastoid aerations, but a good correlation between the sizes of the ipsilateral frontal and maxillary sinuses [6]. They later proposed that the PNS developed by a mutual process of pneumatization, whereas the mastoid air cell system pair may develop by a different process [6]. Lee et al., in their study on the pediatric group, reported that there was no interaction between the three PNS (frontal, maxillary and sphenoid) and mastoid pneumatization, and that both PNS and mastoid pneumatizations were affected by age and had a significant linear regression relationship with age [1]. These authors also stated that paranasal sinus volumes were not different between men and women, however in our study the sphenoid sinus volume was found to be statistically larger in men (p = 0.024).

In their study where they evaluated sinus pathologies in COM and otitis media with cholesteatoma, Arai et al. showed that the coexistence of chronic sinusitis was high in ears with cholesteatoma and that there was a relationship between the presence of cholesteatoma, sphenoid length and Vidic classification score [7]. Kim et al. analyzed the correlation between paranasal sinuses and mastoid air cells using 3D reconstruction of CT images and reported that the only sinus system with a volumetric correlation with mastoid air cells was the sphenoid sinus [4].

Hindi et al. evaluated CT images according to ethnicity, reported a positive correlation between pneumatization of mastoid air cells and pneumatization of the sphenoid sinus [2]. We may say that our results are similar with other studies, however we cannot comment that the sphenoid sinus volume is correlated with mastoid pneumatization. On the contrary, we may say that the contralateral sphenoid volume was larger in our study.

Many studies examining mastoid pneumatization reported that nasal septal deviation and nasal ventilation disorders affected mastoid pneumatization [14, 15]. Maier et al. stated that surgical correction of serious nasal pathologies before tympanoplasty may be beneficial [14]. In another study, Koch et al. concluded that negative middle ear pressure could be corrected by nasal surgery in patients with nasal ventilation disorders [15]. Studies conducted on pediatric cases reported that nasal congestion affected middle ear pressure negatively and increased the incidence of ear pathologies [16, 17]. Yüksel et al. also reported a significantly greater MAC volume on the side with isolated concha bullosa and argued that nasal airflow influences mastoid pneumatization [18].

The relationship between middle ear effusion and Eustachian tube dysfunction has been put forward, and it has been stated that Eustachian tube dysfunction is the main risk factor for benign middle ear disorders. However, it has been argued that nasal and paranasal pathologies and anatomical variations that cause chronic sinonasal inflammation also affect the middle ear mucosa. However, we may state that the publications are not clear regarding the effects of middle ear pathologies on the paranasal sinuses. The main idea advocated in studies involving co-existing pathologies is that the Eustachian tube is affected. However, in our study, we looked at this phenomenon in reverse, and evaluated isolated unilateral ear pathologies in terms of the volumetric dimensions of the mastoid and paranasal sinuses. While hypopneumatization of the mastoid cavity was together with a decrease in the frontal and maxillary sinus volumes compared to the opposite side, we found that the sphenoid sinus volume was larger on the affected side compared to the opposite side; however these results were not statistically significant. Considering that a pathology in the mastoid is necessary during the development of for mastoid for mastoid hypopneumatization, we may comment that it does not have a significant effect on the paranasal sinus development. Here, we only examined the patients in whom we performed type-1 tympanoplasty and did not include more complex cases such as cholesteatoma or polyps due to the multifactorial nature of these pathologies. Increasing the extent of pathology in the middle ear may have a greater impact on the developmental process of the paranasal sinuses.

In general, the small number of patients and the retrospective nature of our study may be regarded as the limitations of our study. In addition, the possibility of subclinical contralateral dysfunction, the lack of groups with different ear pathologies, and the absence of a tool for scoring middle ear mucosa may be considered limitations. However, we suppose that this study will provide useful information for further studies due to its importance in paranasal sinus and mastoid surgery, and our study is the first that revealed the influence and asymmetry of COM on the paranasal sinuses in 3 dimensions.

## Conclusion

In conclusion, middle ear pathologies are associated with decreased mastoid pneumatization, which could potentially influence ipsilateral paranasal sinuses. Revealing anatomic asymmetries and understanding surgical anatomy are valuable for preventing surgical complications. Existing pathologies should warn the surgeon before paranasal or mastoid surgery. Further research in this area will be helpful for understanding the normal and pathological conditions of the paranasal sinuses and mastoid air cells.

## Data Availability

The data are available from the corresponding author upon reasonable request.
